# Clusters and their social determinants associated with vaccination against human papillomavirus in the state of Minas Gerais, Brazil

**DOI:** 10.1590/1980-549720260009

**Published:** 2026-03-16

**Authors:** Josianne Dias Gusmão, Thales Philipe Rodrigues da Silva, Ana Catarina de Araújo Melo, Monica Levi, Jéssica Emanuela de Sena Gonçalves, Bianca Maria Oliveira Lovisaro, Fernanda Penido Matozinhos

**Affiliations:** I Universidade Federal de Minas Gerais, Escola de Enfermagem, Programa de Pós-graduação em Enfermagem - Belo Horizonte (MG), Brazil.; II Secretaria de Estado de Saúde de Minas Gerais - Belo Horizonte (MG), Brazil.; III Universidade Federal de Minas Gerais, Faculdade de Medicina - Belo Horizonte (MG), Brazil.; IV Ministério da Saúde, Coordenação-Geral de Incorporação Científica e Imunização no Programa Nacional de Imunizações - Brasília (DF), Brazil.; V Sociedade Brasileira de Pediatria - São Paulo (SP), Brazil.

**Keywords:** Papillomavirus vaccines, Adolescent, Social determinants of health, Vaccination coverage, Vaccination

## Abstract

**Objective:**

To analyze the clusters and their social determinants associated with vaccination against human papillomavirus (HPV) in the State of Minas Gerais, Brazil.

**Methods:**

Ecological study, carried out in 853 municipalities of Minas Gerais, Brazil. The K-means algorithm was used for clustering analysis. The calculation of vaccination coverage (VC) of the HPV vaccine, for males and females, age group 9 to 14 years, between 2014 and 2022, and socioeconomic indicators were considered.

**Results:**

Males had the lowest proportion of municipalities in Minas Gerais achieving the VC target. Of the 5 Clusters generated, Cluster 1 (259 municipalities) showed high urbanization and high VC, but with challenges for male adherence. Cluster 2 (178 municipalities) identified low VC and high poverty, with limited access to health. Cluster 3 (166 municipalities) presented high VC and lower vulnerability, consisting of municipalities with high vaccination uptake and socioeconomic conditions, indicating effective health policies. In Cluster 4 (166 municipalities), public health weaknesses and the need for targeted interventions were identified. Cluster 5 (84 municipalities) presented high urbanization and inequality, but with a large disparity between urbanization and vaccination adherence.

**Conclusion:**

Thus, this study identified that high urbanization, lower vulnerability and greater access to health services are factors that can positively impact vaccine adherence by adolescents.

## INTRODUCTION

The carcinogenic role of human papillomavirus (HPV) in the development of malignant tumors, such as cervical cancer, anogenital tract cancer, and head and neck cancer, is scientifically proven and recognized worldwide, with primary prevention occurring through HPV vaccines^1^ ^,^ ^2^.

The World Health Organization (WHO) recommended the implementation of HPV vaccination programs around the globe. The first country to introduce the HPV vaccine into its immunization program was Australia, followed by the United States, Canada, and the United Kingdom in 2006. Subsequently, several European countries also introduced the HPV vaccine^3^. However, the HPV vaccine has faced challenges such as changes in the target population and the vaccination schedule over time. Currently, the target audience for this vaccine is adolescents of both female and male sexes, and the age range for HPV vaccination is between 9 and 14 years, with variations in this window across different immunization programs. However, in any country worldwide, the target audience remains under 15 years of age^2^.

Public concern, as well as the concern of the target audience and their guardians, regarding adverse reactions to vaccines has threatened their introduction and successful coverage rates in some countries. Several factors associated with little knowledge and attitudes towards HPV vaccination, such as low parental education levels, lack of participation in health education activities about HPV, low perception of disease risk, and limited knowledge of the efficacy and safety of HPV vaccines, have been documented as reasons for non-adherence (or low adherence) to immunization^2^ ^,^ ^4^ ^,^ ^5^.

In this context, population analyses using aggregated data contribute to the understanding of the social or structural determinants associated with vaccination coverage (VC) in a geographical area, especially when it involves a specific population, such as the target group for HPV vaccination^5^ ^,^ ^6^ ^,^ ^7^.

Thus, family conditions and access to public services serve as indicators of the socioeconomic status of a location, allowing for inferences such as: more urban municipalities have a higher chance of achieving the desired coverage since they provide greater dissemination of information about vaccination campaigns, and also offer more structured health services, such as vaccine availability^5^ ^,^ ^7^.

In light of the above, the objective of this study was to analyze the possible clusters and their social determinants associated with HPV vaccination in the state of Minas Gerais, Brazil.

## METHODS

### Design

This is an epidemiological study with an ecological design and spatial analysis using aggregated historical data, with the units of analysis being the 853 municipalities in the state of Minas Gerais, Brazil. The analysis aims to identify patterns of clustering (clusters) of municipalities with similar characteristics in terms of HPV VC and associated socioeconomic factors.

The state of Minas Gerais consists of 853 municipalities, located in an area of 586,513.984 km^2^, with a population of 20,539,989 inhabitants in the year 2022 and an urbanized area of 4,699.69 km^2^ ^8^. Its territory is divided into 19 Regional Health Superintendencies and nine Regional Health Management offices, which are responsible for coordinating health policies and actions within their areas of coverage^9^ ^,^ ^10^.

It is noteworthy that the units of analysis in this study were the aggregated VC of municipalities in the state of Minas Gerais for adolescents aged 9 to 14 who received the first and second doses of the HPV vaccine from 2014 to 2022. Variables related to socioeconomic indicators and access to health care were considered (Chart 1).

**Chart 1.** Description of variables related to vaccination coverage, socioeconomic indicators, access to health care, urbanization rates, and geographic variables among adolescents residing in municipalities in the state of Minas Gerais from 2014 to 2022.

Indicators related to vaccination coverage, socioeconomic indicators, and geographic variables	Description of variables	Data source
Vaccination coverage (VC)	Proportion of vaccinated male and female adolescents with the 1st and 2nd doses. Until the year 2023, the target was 80%; starting in 2024, it increased to 90% when a single-dose scheme was adopted ^12^.	National Immunization Program Information System (SI-PNI). Population estimates were calculated based on data from the Brazilian Institute of Geography and Statistics (IBGE) and adjusted by the Secretariat of Health Surveillance and Environment.
Sociodemographic indicators, access to health care, and urbanization rate	Percentage of the population living in poverty in the Unified Registry; number of families with an income of up to half the minimum wage; proportion of the population covered by the Family Health Strategy (FHS); urbanization rate (%).	Brazilian Institute of Geography and Statistics (IBGE) and João Pinheiro Foundation.
Geographic variables	Territorial area of the municipalities (in km^2^)	Brazilian Institute of Geography and Statistics (IBGE).

*Municipalities with very low vaccination coverage (0-49%), low (50-79%), and adequate (≥80%).

For the calculation of VC, data were extracted from the National Immunization Program Information System (SI-PNI) using a time frame between 2014 and 2022, and the socioeconomic indicators were obtained from the Brazilian Institute of Geography and Statistics (IBGE) and the João Pinheiro Foundation. Population estimates were calculated based on data from IBGE and adjusted by the Secretariat of Health Surveillance and Environment.

The Ministry of Health has adopted the accumulated method for evaluating the vaccination coverage (VC) of the HPV vaccine since its implementation in 2014, using the cumulative approach for both the first dose (D1) and second dose (D2) in the numerator, and for the denominator, the population recommended for vaccination. In the numerator, the doses administered, recorded in the National Immunization Program Information System (SIPNI), were included according to age and sex. In the denominator, the population of girls and boys recommended for vaccination each year was included, accumulated since the vaccine’s implementation year (2014), estimated by the Ministry of Health (2000 to 2021 - preliminary estimates prepared by the Ministry of Health/SVSA/DAENT/CGIAE), by sex and age group. This method for monitoring HPV vaccination coverage was adopted until the year 2023^11^.

This study considered a two-dose scheme of the HPV vaccine for the age group of 9 to 14 years, with a VC target of 80%, as recommended by the Ministry of Health until the year 2023. The municipalities were classified based on vaccination coverage as very low (0-49%), low (50-79%), and adequate (≥80%)^12^.

Spatial analysis was conducted to identify and evaluate the clusters of vaccinated individuals and possible social factors associated with HPV vaccination coverage. The K-means^13^ algorithm was applied to group municipalities based on similar characteristics related to vaccination coverage and socioeconomic indicators. To minimize the influence of municipalities with extremely low or high populations on the K-means centroids, all continuous variables used in the analysis were normalized. Normalization standardizes the scale of the variables and reduces the impact of extreme values in the distance calculations.

To determine the ideal number of clusters (k), the Elbow Method ^14^, was used, which evaluates the sum of squared distances within each cluster.

Each cluster was analyzed in relation to socioeconomic indicators (percentage of the population living in poverty in the Unified Registry, number of families with an income of up to half the minimum wage), access to health care (proportion of the population covered by the Family Health Strategy [FHS]), urbanization rate [%], and VC). Descriptive tables were generated to compare the means and standard deviations of the variables in each cluster. For spatial statistical analysis, Stata version 16.0 and GeoDa version 1.20.0.8 software were used to represent the geographic distribution of the clusters in the state of Minas Gerais, Brazil.

### Ethical aspects

This study is part of a larger project entitled *Evaluation of Action Research for Increasing Vaccination Coverage among Adolescents in the State of Minas Gerais, Brazil*, approved by the Research Ethics Committee (CEP), in accordance with resolution 466/2012 of the National Health Council^15^, Certificate of Presentation for Ethical Review (CAAE) 69731923.1.0000.5149.

## Data availability statement:

All datasets supporting the results of this study are available upon request to the corresponding author.

## RESULTS

This study considered a two-dose scheme of the HPV vaccine for the age group of 9 to 14 years, with a vaccination coverage target of 80%, as recommended by the Ministry of Health until the year 2023. It is noteworthy that, in 2024, the Ministry of Health recommended a single dose of the HPV vaccine for the age group of nine to 14 years, with a vaccination coverage target of 90%^12^.

**Figure 1.**
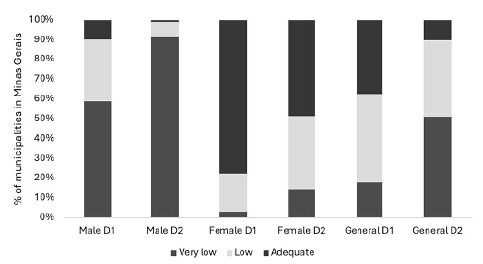
Percentage of municipalities with vaccination coverage for the HPV vaccine (1^st^ and 2^nd^ doses - D1 and D2), Minas Gerais, 2014 - 2022*.

It was found that, for male adolescents, 9.73% of municipalities in Minas Gerais achieved the vaccination coverage target (>80%) for the first dose of the HPV vaccine, and only 0.82% reached this target for the second dose. For female adolescents, 77.49% of municipalities achieved the target for the first dose and 48.53% for the second dose. Regarding overall coverage, it was noted that, for the first dose, 37.51% of municipalities in Minas Gerais achieved adequate coverage, and for the second dose (D2), only 9.96% (Figure 1).

Table 1 shows that the average municipal area was 687.6 km², with an average target male population for the second dose of 1,120 and a female population of 1,070. The average number of families with an income of half the minimum wage was 2,312, and the average percentage of the population living in poverty in the Unified Registry was 26.5%. The average proportion of the population served by the FHS was 92.5%, and the urbanization rate was 77.4% (Table 1).

**Table 1.** Description of socioeconomic variables, urbanization, and access to health care, Minas Gerais, Brazil, 2022.

Variables	Mean	Deviation	Minimum	Q1	Median	Q3	Maximum
Area (km^2^)	687.6	965.8	3.6	196.7	363.8	729.8	10,727,1
Average number of families with an income of half the minimum wage	2,312,0	5,692,0	78.0	664.0	1,140,0	2,135,0	122,308,0
Average percentage of the poor population in the Unified Registration	26.5	13.6	3.3	15.3	23.8	36.3	73.2
Average proportion of the population served by the Family Health Strategy	92.5	14.3	27.8	91.3	100.0	100.0	100.0
Urbanization rate	77.4	14.8	19.6	66.7	78.3	90.6	100.0

**Figure 2.**
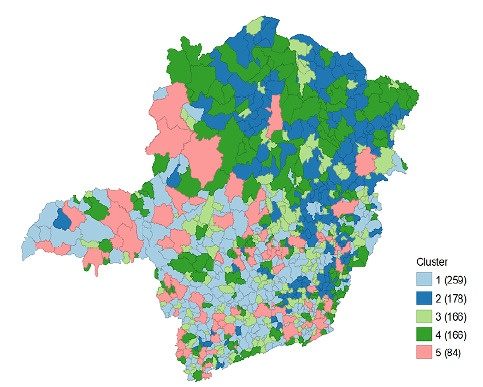
Spatial distribution of vaccination coverage (VC) of the vaccine against human papillomavirus (HPV), socioeconomic indicators, access to health and urbanization (clusters C1 to C5), Minas Gerais. 2022*.

The analysis using K-means generated five clusters (C1 to C5) (Table 2 and Figure 2), which included group municipalities with similar characteristics regarding VC, socioeconomic indicators, urbanization, and access to health care (proportion of the population covered by the FHS). Cluster 1 consisted of 259 municipalities with advanced urbanization and relatively balanced VC, but with challenges for male adherence. The average VC for the first dose was 59.5% for boys, 98.8% for girls, and an overall coverage of 78.4%. When analyzing the second dose, these numbers drop dramatically; 42.4% for boys and 82.1% for girls, resulting in an overall average of 61.6%

**Table 2.** Description of clusters per área, vaccination coverage of the vaccine against the human papillomavirus (HPV) in boys and girls, socioeconomic variables, access to health and urbanization, Minas Gerais, Brazil.

Cluster	Area (km²)	Boys		Girls		General		N. of Families with na income of half the minimm wage	% Poor population in the Unified Registration	% Population with access to health	Urbanization rate (%)
		D1	D2	D1	D2	D1	D2				
C1	534.525	59.499	42.458	98.7883	82.1461	78.4246	61.5926	1,473,49	18.6255	92.307	85.0282
C2	702.186	56.2631	42.4495	95.1109	81.789	74.8643	61.3177	1,665,11	39.5348	98.9212	63.9725
C3	399.745	80.0405	59.3445	122.162	107.008	100.259	82.2286	938.277	29.8704	98.5846	71.619
C4	908.524	39.4332	27.6849	71.2389	57.1134	54.7961	41.9215	1,905,34	28.2889	96.5353	77.5948
C5	1,260,84	45.7724	31.6746	79.2571	62.9499	62.1113	46.9371	9,788,29	12.6252	59.7048	93.6537

*Cluster (C1 to C5): municipalities with similar characteristics regarding VC of the vaccine against HPV, socioeconomic indicators, access to health and urbanization.

Regarding the environmental variables, the mean territorial area is 534.525 km², with an average of 1,473.49 families earning an average of up to one minimum wage, an average of 18.62% of the population living in poverty, an average of 92.90% with access to the Family Health Strategy (FHS), and an average urbanization rate of 85.02%.

Cluster 2 (Table 2 and Figure 2) consisted of 178 municipalities with low VC, high poverty, and limited access to health care. It showed an average VC below expectations, especially for the first dose for boys (56.3%) and girls (95.1%), with an overall coverage of 74.9%. For the second dose, values were similar to those in Cluster 1, but with a slight decrease in the overall average (61.3%).

Cluster 3 (Table 2 and Figure 2), in turn, was characterized by 166 municipalities with high VC and lower vulnerability, consisting of municipalities with good socioeconomic indicators and high vaccination adherence, indicating effective health policies. Cluster 4 (Table 2 and Figure 2) was characterized by 166 municipalities with weaknesses in public health and a high need for targeted interventions. It showed low VC for boys (39.4%) and girls (71.2%) for the first dose, and for the second dose, the rates were even lower for boys (27.7%) and girls (57.1%).

Finally, Cluster 5 (Table 2 and Figure 2) was composed of 84 urbanized municipalities with high inequality, characterized as highly urbanized municipalities but with a significant disparity between urbanization and vaccination adherence.

Figure 2 shows the spatial distribution of the clusters in the state of Minas Gerais, Brazil.

## DISCUSSION

The results of the cluster analysis on HPV vaccination coverage in Minas Gerais, Brazil, revealed significant disparities in vaccination adherence among different population groups and regions of the state. These findings reflect the complex interaction between socioeconomic, geographic, and structural factors that directly influence the HPV vaccination coverage goal.

Male VC shows the lowest rates, with only 9.73% of municipalities reaching the target for the first dose and 0.82% for the second dose. For girls, although the rates are better, there are still significant gaps, with 77.49% and 48.53% of municipalities reaching the target for the first and second doses, respectively.

Regarding the analysis of the clusters (C1 to C5), this study found that Cluster 1 revealed municipalities with high urbanization and relatively balanced VC, but with challenges in HPV vaccine adherence among boys. This discrepancy between the sexes may be related to cultural factors and the perception of HPV risk, which is traditionally associated with female health. Authors confirm that campaigns aimed at the male audience have less reach due to the perceived lower prioritization of this vaccine for boys^16^.

A study conducted in Brazil that evaluated HPV vaccination coverage among boys from 2017 to 2022 identified a decrease in the uptake of vaccination among boys during the assessed period, leading to VC rates well below the targets set by the National Immunization Program (PNI)^17^. The lack of educational programs targeted at boys and their families is one of the factors contributing to the decreased intention to vaccinate and highlights the need for interventions that consider cultural and gender barriers to increase vaccination adherence^18^.

An integrative review identified that girls tend to get vaccinated against HPV more than boys, and the dissemination of information about the consequences of HPV in females, such as cervical cancer, may have contributed to the lack of awareness regarding the availability of the vaccine for both sexes, resulting in lower vaccination coverage among male adolescents^19^. Furthermore, a study on HPV VC in the Northeast region of Brazil from 2013 to 2021 identified that VC for males was lower than for females in all states and for both doses^20^.

In this study, Cluster 2 identified municipalities with low VC and high poverty, with limited access to health care and low adherence to vaccination campaigns. Cluster 4 also showed low adherence and structural weaknesses. The low VC may be associated with logistical barriers and access to health care, which are widely discussed in the literature on health inequalities^21^. A study that evaluated HPV VC in Brazil identified that social inequalities can lead to inequity in access to HPV vaccination, such as lack of urbanization, poor income distribution, and lack of access to health services, interfering with development and directly harming the health status of adolescents^22^. Another study found similar results, showing that individuals living in more deprived areas had a lower likelihood of starting and completing the HPV vaccination schedule ^23^.

Cluster 3, composed of municipalities with high VC and lower social vulnerability, confirms the importance of favorable socioeconomic indicators, high urbanization rates, and efficient access to the FHS. An analysis of HPV VC conducted in the state of Paraná, Brazil, identified a positive relationship between VC, socioeconomic indicators, and income; thus, the higher the socioeconomic level of the population, the better the health indicators^24^. Consistent with these findings, a study in the United States identified that in areas with access to routine health care, individuals aged 9 to 26 who were eligible for HPV vaccination were more likely to start the vaccination schedule^25^.

Cluster 5 included urbanized municipalities, but with high social inequality and low VC. Despite urbanization, the absence of effective public policies for vulnerable groups results in low vaccination rates. This outcome reaffirms that families most affected by the lack of infrastructure and access to health care may reside in cities in slum areas or metropolitan regions^26^. Regarding urbanization, a study conducted in the United States identified a lower likelihood of HPV vaccination in suburban and rural areas ^7^. A study in Forlì, northern Italy, found that the presence of a vaccination center in less urbanized areas significantly increased the likelihood of acceptance of all available vaccines compared to areas without a vaccination center or in more urbanized areas that have a vaccination center^27^.

The results of the cluster analysis underscore significant disparities in HPV VC in Minas Gerais, highlighting that socioeconomic factors, access to health care, urbanization, and information acquisition play determining roles in adherence to the HPV vaccine. In this sense, the process of urbanization, coupled with an individual’s improved socioeconomic status, facilitates greater access to health services. It also enables better access to information about the importance of preventing HPV-related infections and the benefits of vaccination, which consequently reflects in higher vaccination adherence and increased coverage.

In this study, a lower percentage of municipalities in the state of Minas Gerais was found to reach the VC target for the HPV vaccine among males. It was also identified that higher urbanization, lower social vulnerability, and greater access to health services are factors that can positively impact adolescents’ adherence to the HPV vaccine.

Although there have been improvements in some municipalities with more favorable conditions, the low vaccination coverage among boys, the discrepancy between doses, and the structural weaknesses in public health in vulnerable areas highlight the urgent need for targeted interventions for the eligible population for HPV vaccination, taking into account social determinants.

The greater vulnerability observed in clusters 4 and 5 revealed the influence of social and logistical barriers, especially in regions with larger territorial extent and lower population density. Conversely, the positive results in clusters 1 and 3 reinforce that investments in health infrastructure, social policies, and community mobilization strategies can significantly increase VC.

Based on this evidence, it is recommended to prioritize vulnerable municipalities in awareness campaigns, improve vaccination logistics, and expand the FHS in remote areas. Additionally, continuous monitoring of vaccine hesitancy and the development of public policies that integrate educational actions, reduce social inequalities, and ensure universal access to vaccination are essential.

In this study, the main limitations are related to the use of secondary data for calculating HPV VC, socioeconomic indicators, access to health care, and urbanization rates. Furthermore, as this is an ecological study, the findings do not necessarily imply that the same association occurs at the individual level. Nevertheless, the importance of using a rigorous methodology is emphasized, along with the significance of employing spatial multivariate models and research that relates VC to socioeconomic indicators and geographic variables, facilitating better planning of targeted actions to regain vaccination coverage.

It can be concluded that the results of this study reinforce the importance of intersectoral approaches to ensure health equity, contributing to the expansion of HPV VC and the reduction of related disease incidence in the state of Minas Gerais, Brazil.

## References

[1] Canfell K, Kim JJ, Brisson M, Keane A, Simms KT, Caruana M (2020). Mortality impact of achieving WHO cervical cancer elimination targets: a comparative modelling analysis in 78 low-income and lower-middle-income countries. Lancet.

[2] World Health Organization (2023). Immunization coverage.

[3] Markowitz LE, Tsu V, Deeks SL, Cubie H, Wang SA, Vicari AS (2012). Human papillomavirus vaccine introduction--the first five years. Vaccine.

[4] Lee A, Wong MC, Chan TT, Chan PK (2015). A home-school-doctor model to break the barriers for uptake of human papillomavirus vaccine. BMC Public Health.

[5] Moura L de L, Codeço CT, Luz PM (2021). Human papillomavirus (HPV) vaccination coverage in Brazil: Spatial and age cohort heterogeneity. Rev Bras Epidemiol.

[6] Patel C, Brotherton JM, Pillsbury A, Jayasinghe S, Donovan B, Macartney K (2018). The impact of 10 years of human papillomavirus (HPV) vaccination in Australia: what additional disease burden will a nonavalent vaccine prevent?. Euro Surveill.

[7] Williams CL, Walker TY, Elam-Evans LD, Yankey D, Fredua B, Saraiya M (2020). Factors associated with not receiving HPV vaccine among adolescents by metropolitan statistical area status, United States, National Immunization Survey-Teen, 2016-2017. Hum Vacc Immunother.

[8] Instituto Brasileiro de Geografia e Estatística (2023). Minas Gerais.

[9] Minas Gerais (2023). Decreto Estadual nº 48.661, de 31 de julho de 2023. Dispõe sobre a organização da Secretaria de Estado de Saúde.

[10] Minas Gerais (2024). Superintendências Regionais de Saúde (SRS) e Gerências Regionais de Saúde (GRS).

[11] Brasil. Ministério da Saúde (2025). Nota Técnica nº 16/2025-DPNI/SVSA/MS. Trata-se de orientação quanto à nova metodologia para avaliação dos indicadores de vacinação contra o HPV utilizando o método de coorte pelo ano de nascimento.

[12] Brasil. Ministério da Saúde. Departamento do Programa Nacional de Imunizações. Coordenação-Geral de Incorporação Ciência e Imunização. Secretaria de Vigilância em Saúde e Ambiente (2024). Nota Técnica nº 41/2024-CGICI/DPNI/SVSA/MS - Atualização das recomendações da vacinação contra HPV no Brasil.

[13] Liu R (2022). Data analysis of educational evaluation using K-means clustering method. Comput Intell Neurosci.

[14] Ashari IF, Nugroho ED, Randi Baraku R, Yanda IN, Liwardana R (2023). Analysis of elbow, silhouette, Davies-Bouldin, Calinski-Harabasz, and Rand-Index evaluation on K-means algorithm for classifying flood-affected areas in Jakarta. J Appl Informatics Comput.

[15] Conselho Nacional de Saúde (2012). Resolução Nº 466, de 12 de dezembro de 2012.

[16] Martins LC, Silva AM, Oliveira FR (2020). Fatores associados à hesitação vacinal em adolescentes: um olhar sobre a vacina contra o HPV no Brasil. Rev Bras Saúde Materno Infantil.

[17] Grossi L, dos Santos Oliveira GM, de Oliveira Lacerda Raposo V, de Sá Mota DM (2024). Aspectos da vacinação contra HPV em meninos brasileiros entre os anos de 2017 a 2022: um estudo ecológico. Braz J Implantol Health Sci.

[18] Cho YH, Kim TI (2024). A six-week smartphone-based program for HPV prevention among mothers of school-aged boys: a quasi-experimental study in South Korea. Healthcare.

[19] Silva MS da, Sáfadi MAP, Silva FL da, Bezerra AMF, Nascimento ARS do, Gondim JB (2024). Social determinants of health in adherence to HPV vaccination among adolescents: an integrative review. Res Soc Dev.

[20] Glehn MDPV, Nascimento LMD, Freire KMR, Minuzzi TTC, Hott CE, Maranhão AGK (2023). Cobertura da vacinação contra papilomavírus humano no Nordeste do Brasil, 2013-2021: estudo descritivo. Epidemiol Serv Saúde.

[21] Macedo TRO, Borges MFSO, Silva IF, França AP, Moraes JC (2024). Cobertura vacinal, barreiras e hesitação vacinal em crianças de até 24 meses: inquérito populacional em uma capital do oeste amazônico. Epidemiol Serv Saúde.

[22] Castro BT de, Quaresma ALP, Azevêdo AO, Silva LM da, Teixeira CSS (2022). Coverage of doses of the HPV vaccine and variation by level of material deprivation in Brazilian municipalities, 2012 to 2018. Res Soc Dev.

[23] Kurani S, MacLaughlin KL, Jacobson RM, St Sauver JL, Jenkins GD, Fan C (2022). Socioeconomic disadvantage and human papillomavirus (HPV) vaccination uptake. Vaccine.

[24] Pelloso FC, Pazin DC, Silva LL, Carvalho MDdB, Borghesan DHP, Consolaro MEL (2024). Cobertura vacinal contra o HPV no estado do Paraná: distribuição espacial e avanços em saúde pública. Vaccines.

[25] Goel K, Vasudevan L (2021). Disparities in healthcare access and utilization and human papillomavirus (HPV) vaccine initiation in the United States. Hum Vaccin Immunother.

[26] Santos TM, Cata-Preta BO, Wendt A. (2024). Exploring the “Urban Advantage” in Access to Immunization Services: A Comparison of Zero-Dose Prevalence Between Rural, and Poor and Non-poor Urban Households Across 97 Low- and Middle-Income Countries. J Urban Health.

[27] Ceccarelli A, Soro G, Reali C, Biguzzi E, Farneti R, Frassineti V (2024). The influence of altitude, urbanization, and local vaccination centers on vaccine uptake within an Italian health district: an analysis of 15,000 individuals eligible for vaccination. Vaccines.

