# Supplementary material for: Clusters and their social determinants associated with vaccination against human papillomavirus in the state of Minas Gerais, Brazil
Source: Rev Bras Epidemiol. 2026 Mar 16;29:e260009. doi: 10.1590/1980-549720260009 (PMC12995341; doi:10.1590/1980-549720260009)
Supplement: Translation

# Translation: Clusters e seus determinantes sociais associados à vacinação contra o papilomavírus humano no estado de Minas Gerais, Brasil

**DOI:** 10.1590/1980-549720260009.2

**Authors:** Josianne Dias Gusmão, Thales Philipe Rodrigues da Silva, Ana Catarina de Araújo Melo, Monica Levi, Jéssica Emanuela de Sena Gonçalves, Bianca Maria Oliveira Lovisaro, Fernanda Penido Matozinhos

**Affiliations:** I Universidade Federal de Minas Gerais, Escola de Enfermagem, Programa de Pós-graduação em Enfermagem - Belo Horizonte (MG), Brasil.; II Secretaria de Estado de Saúde de Minas Gerais - Belo Horizonte (MG), Brasil.; III Universidade Federal de Minas Gerais, Faculdade de Medicina - Belo Horizonte (MG), Brasil.; IV Ministério da Saúde, Coordenação-Geral de Incorporação Científica e Imunização no Programa Nacional de Imunizações - Brasília (DF), Brasil.; V Sociedade Brasileira de Pediatria - São Paulo (SP), Brasil.

**Keywords:** Vacinas contra papilomavirus, Adolescente, Determinantes sociais da saúde, Cobertura vacinal, Vacinação

## Abstract

**Objetivo:**

Analisar os *clusters* e seus determinantes sociais associados à vacinação contra o papilomavírus humano (HPV) no estado de Minas Gerais, Brasil.

**Métodos:**

Estudo ecológico, realizado nos 853 municípios de Minas Gerais, Brasil. O algoritmo K-means foi utilizado para a análise dos *clustering.* Considerou-se o cálculo da cobertura vacinal (CV) da vacina HPV, para o público masculino e feminino, faixa etária de nove a 14 anos, entre 2014 e 2022, e os indicadores socioeconômicos.

**Resultados:**

O sexo masculino apresentou a menor proporção de municípios de Minas Gerais com alcance de meta de CV. Dos cinco *clusters* gerados, o 1 (259 municípios) apresentou alta urbanização e CV alta, mas com desafios para a adesão masculina. O *cluster* 2 (178 municípios), identificou baixa CV e alta pobreza, com acesso limitado à saúde. O *cluster* 3 (166 municípios) apresentou alta CV e menor vulnerabilidade, sendo constituído por municípios com elevada adesão vacinal e condições socioeconômicas, indicando políticas de saúde efetivas. No *cluster* 4 (166 municípios), foram identificadas fragilidades em saúde pública e necessidade de intervenções direcionadas. O *cluster* 5 (84 municípios) apresentou alto urbanismo e desigualdade, mas com grande disparidade entre urbanização e adesão à vacinação.

**Conclusão:**

Assim, este estudo identificou que alta urbanização, menor vulnerabilidade e maior acesso aos serviços de saúde são fatores que podem impactar positivamente a adesão à vacina pelos adolescentes.

## INTRODUÇÃO

O papel carcinogênico do papilomavírus humano (HPV) no desenvolvimento de tumores malignos, como o câncer cervical, do trato anogenital e de cabeça e pescoço, é cientificamente comprovado e reconhecido mundialmente, e a prevenção primária ocorre por meio das vacinas contra o HPV^1^ ^,^ ^2^.

A Organização Mundial da Saúde (OMS) recomendou a implementação de programas com vacinação contra o HPV em todo o mundo. O primeiro país a introduzir a vacina HPV em programa de imunização foi a Austrália, em seguida EUA, Canadá e Reino Unido, em 2006. Na sequência vários países europeus também introduziram a vacina HPV^3^. Entretanto, a vacina contra o HPV tem enfrentado desafios, como mudanças na população alvo e no esquema de vacinação ao longo do tempo. Atualmente, o público alvo dessa vacina são os adolescentes dos sexos feminino e do masculino, e a faixa etária para a vacinação contra HPV é entre nove e 14 anos de idade, com variações nessa janela nos diversos programas de imunização, mas em qualquer país do mundo o público-alvo fica abaixo dos 15 anos de idade^2^.

A preocupação pública e também do público-alvo, assim como de seus responsáveis, com relação às reações adversas às vacinas ameaçou a sua introdução e as taxas de coberturas bem-sucedidas em alguns países. Vários fatores associados ao baixo conhecimento e às atitudes com relação à vacinação contra o HPV, como o baixo nível de escolaridade dos pais, a não participação em atividades de educação em saúde sobre o HPV, a baixa percepção de risco da doença e um conhecimento limitado da eficácia e segurança das vacinas HPV, foram documentados como motivos para a não (ou baixa) adesão à imunização^2^ ^,^ ^4^ ^,^ ^5^.

Nesse contexto, as análises populacionais, com dados agregados, contribuem para a compreensão dos determinantes sociais ou estruturais ligados à CV numa área geográfica, principalmente quando se trata de uma população específica, como a do grupo alvo da vacinação contra o HPV^5^ ^,^ ^6^ ^,^ ^7^.

Assim, as condições do agregado familiar e o acesso a serviços públicos são como indicadores de *status* socioeconômico de um local, possibilitando inferências como: municípios mais urbanos apresentarem maiores chances de alcançar a cobertura desejada, pois têm maior divulgação de informações sobre campanhas de vacinação e também oferecem serviços de saúde mais estruturados, como o de oferta de vacinas^5^ ^,^ ^7^.

Diante do exposto, o objetivo deste estudo foi analisar os possíveis *clusters* e seus determinantes sociais associados à vacinação contra o HPV no estado de Minas Gerais, Brasil.

## MÉTODOS

### Delineamento

Trata-se de estudo epidemiológico com delineamento ecológico e análise espacial com dados históricos agregados, tendo como unidades de análise os 853 municípios do estado de Minas Gerais, Brasil. A análise objetiva identificar padrões de agrupamento (*clusters*) de municípios com características semelhantes em termos de CV contra o HPV e fatores socioeconômicos associados.

O estado de Minas Gerais é constituído por 853 municípios, localizados em uma área de 586.513,984 km^2^, com população de 20.539.989 habitantes no ano de 2022 e área urbanizada de 4.699,69 km^2^ ^8^. O seu território é dividido em 19 Superintendências Regionais de Saúde e nove Gerências Regionais de Saúde, que têm como atribuição a coordenação das políticas e ações de saúde da área de abrangência^9^ ^,^ ^10^.

Ressalta-se que as unidades de análise deste estudo foram as coberturas vacinais agregadas dos municípios do estado de Minas Gerais, de adolescentes de nove a 14 anos de idade, vacinados com a primeira e segunda doses da vacina contra o HPV no período de 2014 a 2022. Consideraram-se variáveis relacionadas aos indicadores socioeconômicos e de acesso à saúde (Quadro 1).

**Quadro 1.** Descrição das variáveis relacionadas a cobertura vacinal, indicadores socioeconômicos, acesso à saúde, taxa de urbanização e variáveis geográficas .entre os adolescentes residentes nos municípios do estado de Minas Gerais, nos anos de 2014 a 2022.

Indicadores relacionados a cobertura vacinal, indicadores socioeconômicas e variáveis geográficas	Descrição das variáveis	Fonte de dados
Cobertura vacinal (CV)	Proporção de adolescentes do sexo masculino e feminino vacinados com a 1ª e 2ª doses. Até o ano de 2023 a meta era de 80%; a partir de 2024, passou para 90%, quando foi adotado o esquema de dose única^12^.	Sistema de Informação do Programa Nacional de Imunizações (SI-PNI). As estimativas populacionais foram calculadas com base no IBGE e ajustadas pela Secretaria de Vigilância em Saúde e Ambiente.
Indicadores socioeconômicos, acesso à saúde e taxa de urbanização	Percentual da população pobre no Cadastro Único; número de famílias com renda de até meio salário-mínimo; proporção da população coberta pela Estratégia de Saúde da Família (ESF); taxa de urbanização (%).	Instituto Brasileiro de Geografia e Estatística (IBGE) e Fundação João Pinheiro.
Variáveis geográficas	Área territorial dos municípios (em km²).	Instituto Brasileiro de Geografia e Estatística (IBGE).

*Municípios com cobertura vacinal muito baixa (0-59%), baixa (60-79%) e adequada (≥80%).

Para o cálculo da CV foram extraídos os dados do Sistema de Informação do Programa Nacional de Imunizações (SI-PNI) utilizando um recorte temporal entre 2014 e 2022, e os indicadores socioeconômicos foram obtidos do Instituto Brasileiro de Geografia e Estatística (IBGE) e da Fundação João Pinheiro. As estimativas populacionais foram calculadas com base no IBGE e ajustadas pela Secretaria de Vigilância em Saúde e Ambiente.

O Ministério da Saúde adotou como metodologia de avaliação da CV da vacina contra o HPV, desde a sua implantação no ano de 2014, a forma acumulada, tanto para a primeira dose (D1) como para segunda dose (D2) no numerador e, para o denominador, a população com recomendação de vacinação. No numerador, foram incluídas as doses aplicadas, disponibilizadas no Sistema de Informações do Programa Nacional de Imunizações (SIPNI), de acordo com idade e sexo. No denominador, foi incluída a população de meninas e meninos com recomendação de vacinação em cada ano, acumulada desde o ano de implantação da vacina (2014), estimada pelo Ministério da Saúde (2000 a 2021 - Estimativas preliminares elaboradas pelo Ministério da Saúde/SVSA/DAENT/CGIAE), por sexo e faixa etária. E esse método de monitoramento da CV da vacina contra o HPV foi adotado até o ano de 2023^11^.

Considerou-se neste estudo o esquema de duas doses da vacina HPV para a faixa etária de nove a 14 anos, com meta de cobertura vacinal de 80%, conforme recomendação do Ministério da Saúde até o ano de 2023. Os municípios foram classificados com cobertura vacinal muito baixa (0-59%), baixa (60-79%) e adequada (≥80%)^12^.

Realizou-se uma análise espacial para identificar e avaliar os aglomerados (*clusters*) de vacinados e os possíveis fatores sociais associados à cobertura vacinal da vacina contra o HPV. Foi aplicado o algoritmo K-means^13^ para agrupar os municípios com base em características semelhantes relacionadas à cobertura vacinal e aos indicadores socioeconômicos. Para minimizar a influência de municípios com população extremamente baixa ou alta sobre os centroides do K-means, todas as variáveis contínuas utilizadas na análise foram normalizadas. A normalização padroniza a escala das variáveis e reduz a influência de valores extremos nos cálculos de distância.

Para determinar o número ideal de *clusters* (k), utilizou-se o Elbow Method^14^, que avalia a soma das distâncias quadráticas internas de cada *cluster*.

Cada *cluster* foi analisado com relação aos indicadores socioeconômicos (percentual da população pobre no Cadastro Único, número de famílias com renda de até meio salário-mínimo), acesso à saúde (proporção da população coberta pela Estratégia Saúde da Família [ESF]), taxa de urbanização [%] e CV). Foram geradas tabelas descritivas para comparar as médias e desvios padrão das variáveis em cada *cluster*. Para a análise estatística espacial, foram utilizados os *softwares* Stata versão 16.0 e Geoda versão 1.20.0.8. para representar a distribuição geográfica dos *clusters* no estado de Minas Gerais, Brasil.

### Aspectos éticos

Este estudo integra um projeto maior, intitulado *Avaliação da pesquisa-ação para o aumento da cobertura vacinal em adolescentes no estado de Minas Gerais, Brasil*, aprovado pelo Comitê de Ética em Pesquisa (CEP), conforme resolução 466/2012 do Conselho Nacional de Saúde^15^, Certificado de Apresentação para Apreciação Ética (CAAE) 69731923.1.0000.5149.

## Declaração de disponibilidade de dados:

Todo o conjunto de dados que dá suporte aos resultados deste estudo está disponível mediante solicitação ao autor correspondente.

## RESULTADOS

Neste estudo foi considerado o esquema de duas doses da vacina HPV para a faixa etária de nove a 14 anos, com meta de cobertura vacinal de 80%, conforme recomendação do Ministério da Saúde até o ano de 2023. Destaca-se que, no ano de 2024, o Ministério da Saúde recomendou a dose única da vacina HPV para a faixa etária de nove a 14 anos de idade, com meta de cobertura vacinal de 90%^12^.

Constatou-se que, para adolescentes do sexo masculino, 9,73% dos municípios de Minas Gerais alcançaram a meta de cobertura vacinal (>80%) para a primeira dose da vacina contra o HPV, e apenas 0,82% alcançaram essa meta para a segunda dose. Para adolescentes do sexo feminino, 77,49% dos municípios alcançaram a meta para a primeira dose e 48,53% para a segunda dose. Quanto à cobertura geral, observou-se que, para a 1ª dose, 37,51% dos municípios mineiros atingiram cobertura adequada e, para a 2ª dose (D2), somente 9,96% (Figura 1).

**Figura 1.** Percentual de municípios com cobertura vacinal para a vacina HPV (1ª e 2ª doses - D1 e D2), Minas Gerais. 2014 - 2022*.

Observa-se, na Tabela 1, que a área média municipal foi de 687,6 km^2^, com população masculina alvo da segunda dose de 1.120, em média, e população feminina de 1.070. O número médio de famílias com renda de meio salário-mínimo foi de 2.312, e o percentual médio de população pobre no Cadastro Único foi de 26,5%. A proporção média da população atendida pela ESF foi de 92,5%, e a taxa de urbanização foi de 77,4% (Tabela 1).

**Tabela 1.** Descrição das variáveis socioeconômicas, urbanização e acesso à saúde, Minas Gerais, Brasil, 2022.

Variáveis	Média	Desvio	Mínimo	Q1	Mediana	Q3	Máximo
Área (km^2^)	687,6	965,8	3,6	196,7	363,8	729,8	10.727,1
Número médio de famílias com renda de meio salário-mínimo	2.312,0	5.692,0	78,0	664,0	1.140,0	2.135,0	122.308,0
Percentual médio de população pobre no Cadastro Único	26,5	13,6	3,3	15,3	23,8	36,3	73,2
Proporção média da população atendida pela Estratégia Saúde da Família	92,5	14,3	27,8	91,3	100,0	100,0	100,0
Taxa de urbanização	77,4	14,8	19,6	66,7	78,3	90,6	100,0

A análise com K-means gerou cinco *clusters* (C1 a C5) (Tabela 2 e Figura 2), que agrupam municípios com características semelhantes com relação à CV, indicadores socioeconômicos, urbanização e acesso à saúde (proporção da população coberta pela ESF). O *cluster* 1 foi composto de 259 municípios com urbanização avançada e CV relativamente equilibrada, mas com desafios para a adesão masculina. A cobertura vacinal média na 1ª dose para meninos foi de 59,5%, para as meninas foi de 98,8% e cobertura geral de 78,4%. Ao se analisar a 2ª dose, esses números caem de forma dramática; 42,4% para os meninos e 82,1% para as meninas, gerando a média geral de 61,6%.

**Tabela 2.** Descrição dos *clusters* por área, cobertura vacinal da vacina contra o papilomavírus humano (HPV) em meninos e meninas, variáveis socioeconômicas, acesso à saúde e urbanização, Minas Gerais, Brasil.

Cluster	Área (km²)	Meninos		Meninas		Geral		Nº Famílias com renda de meio salário-mínimo	% População pobre no Cadastro Único	% População com acesso à saúde	Taxa de urbaniza-ção (%)
		D1	D2	D1	D2	D1	D2				
C1	534,525	59,499	42,458	98,7883	82,1461	78,4246	61,5926	1.473,49	18,6255	92,307	85,0282
C2	702,186	56,2631	42,4495	95,1109	81,789	74,8643	61,3177	1.665,11	39,5348	98,9212	63,9725
C3	399,745	80,0405	59,3445	122,162	107,008	100,259	82,2286	938,277	29,8704	98,5846	71,619
C4	908,524	39,4332	27,6849	71,2389	57,1134	54,7961	41,9215	1.905,34	28,2889	96,5353	77,5948
C5	1.260,84	45,7724	31,6746	79,2571	62,9499	62,1113	46,9371	9.788,29	12,6252	59,7048	93,6537

*Cluster (C1 a C5): municípios com características semelhantes em relação à CV da vacina contra o HPV, indicadores socioeconômicos, acesso à saúde e urbanização.

**Figura 2.** Distribuição espacial da cobertura vacinal (CV) da vacina contra o papilomavírus humano (HPV), indicadores socioeconômicos, acesso à saúde e urbanização (*clusters* C1 a C5), Minas Gerais. 2022*.

Quanto às variáveis ambientais, a média territorial é de 534,525 km^2^, com média de 1.473,49 famílias com renda média de até um salário-mínimo, média de 18,62% da população em pobreza, média de 92,90% com acesso à ESF e taxa de urbanização média de 85,02%.

O *cluster* 2 (Tabela 2 e Figura 2) foi constituído por 178 municípios com baixa CV, alta pobreza e acesso limitado à saúde. Apresentou CV média abaixo do esperado, especialmente na 1ª dose para meninos (56,3%) e meninas (95,1%), com cobertura geral de 74,9%. Na 2ª dose, observaram-se valores similares ao C1, mas com leve queda na média geral (61,3%).

O *cluster* 3 (Tabela 2 e Figura 2), por sua vez, foi caracterizado por 166 municípios com alta CV e menor vulnerabilidade, sendo constituído por municípios com bons indicadores socioeconômicos e alta adesão vacinal, indicando políticas de saúde efetivas. O *cluster* 4 (Tabela 2 e Figura 2) foi caracterizado por 166 municípios com fragilidades em saúde pública e alta necessidade de intervenções direcionadas. Caracterizou-se por CV baixa para os meninos (39,4%) e meninas (71,2%) na 1ª dose e, na 2ª dose, os índices foram ainda menores nos meninos (27,7%) e nas meninas (57,1%).

Por fim, o *cluster* 5 (Tabela 2 e Figura 2) foi constituído por 84 municípios urbanizados com alta desigualdade, sendo municípios altamente urbanizados, mas com grande disparidade entre urbanização e adesão à vacinação.

Na Figura 2, é apresentada a distribuição espacial dos *clusters* no estado de Minas Gerais, Brasil.

## DISCUSSÃO

Os resultados da análise de *clusters* sobre a CV contra o HPV em Minas Gerais, Brasil revelaram disparidades significativas na adesão vacinal entre os diferentes grupos populacionais e regiões do estado. Esses achados refletem a complexa interação entre fatores socioeconômicos, geográficos e estruturais, que influenciam diretamente na meta de CV da vacina HPV.

A CV masculina apresenta os índices mais baixos, com apenas 9,73% dos municípios atingindo a meta para a primeira dose e 0,82% para a segunda dose. Para as meninas, embora os índices sejam melhores, ainda há lacunas significativas, com 77,49 e 48,53% dos municípios atingindo a meta para a primeira e segunda doses, respectivamente.

Com relação à análise dos *clusters* (C1 a C5), neste estudo, o *cluster* 1 revelou municípios com alta urbanização e CV relativamente equilibrada, mas com desafios para a adesão a vacina HPV dos meninos. Essa disparidade entre os sexos pode estar relacionada a fatores culturais e à percepção de risco de HPV, tradicionalmente associado à saúde feminina. Autores confirmam que as campanhas voltadas ao público masculino têm menor alcance em razão da menor priorização percebida dessa vacina para meninos^16^.

Um estudo, realizado no Brasil, que avaliou a CV da vacina contra HPV nos meninos, no período de 2017 a 2022, identificou que no período avaliado foi identificada redução pela procura da vacinação pelos meninos e, consequentemente, as CV ficaram bem abaixo da meta preconizada pelo Programa Nacional de Imunizações (PNI)^17^. A falta de programas educacionais direcionados para os meninos e as famílias é um dos fatores que contribuem para a redução da intenção de vacinação e sinalizam a necessidade de intervenções que considerem as barreiras culturais e de gênero para aumentar a adesão à vacinação^18^.

Uma revisão integrativa identificou que as meninas costumam se vacinar contra o HPV mais que os meninos, e a divulgação de informações sobre as consequências do HPV no sexo feminino, como o câncer cervical, pode ter contribuído para o desconhecimento da disponibilidade da vacina para ambos os sexos, resultando em menor CV entre os adolescentes do sexo masculino^19^. Ademais, um estudo sobre a CV contra HPV na Região Nordeste do Brasil, no período de 2013 a 2021, identificou que as CV para o sexo masculino foram mais baixas do que para o sexo feminino, em todos os estados e em ambas as doses^20^.

Neste estudo, o *cluster* 2 identificou municípios com baixa CV e alta pobreza, com acesso limitado à saúde e baixa adesão às campanhas vacinais. O *cluster* 4 também apresentou baixa adesão e fragilidades estruturais. A baixa CV pode estar associada a barreiras logísticas e de acesso à saúde, amplamente discutidas na literatura sobre desigualdades em saúde^21^. Um estudo que avaliou a CV contra HPV no Brasil identificou que as desigualdades sociais podem causar iniquidade no acesso à vacinação contra HPV, como falta de urbanização, má distribuição de renda e falta de acesso aos serviços de saúde, interferindo no desenvolvimento e prejudicando diretamente a situação de saúde dos adolescentes^22^. Outro estudo encontrou resultados semelhantes, e indivíduos que viviam em locais mais carentes tiveram menor possibilidade de iniciar e concluir o esquema de vacinação contra o HPV^23^.

O *cluster* 3, composto de municípios com alta CV e menor vulnerabilidade social, confirma a importância de indicadores socioeconômicos favoráveis, alta taxa de urbanização e acesso eficiente à ESF. Uma análise da cobertura vacinal contra HPV realizada no estado do Paraná, Brasil, identificou relação positiva entre CV, indicadores socioeconômicos e renda; assim, quanto maior o nível socioeconômico da população, melhores os indicadores de saúde^24^. Concordando com esses achados, um estudo nos Estados Unidos identificou que, nos locais com acesso a atendimento de saúde de rotina, os indivíduos de nove a 26 anos elegíveis para a vacinação contra HPV tiveram mais possibilidades de iniciar o esquema de vacinação contra o HPV^25^.

O *cluster* 5 contemplou municípios urbanizados, mas com alta desigualdade social e baixa cobertura vacinal. Apesar da urbanização, a ausência de políticas públicas eficazes para grupos vulneráveis resulta em baixos índices de vacinação. Esse resultado reafirma que as famílias mais prejudicadas com a falta de infraestrutura e acesso à saúde podem residir nas cidades em regiões de favelas ou regiões metropolitanas^26^. Quanto à urbanização, um estudo realizado nos Estados Unidos identificou menor probabilidade da vacinação contra o HPV em áreas suburbanas e rurais^7^. Já um estudo em Forlì, norte da Itália, identificou que a presença de um centro de vacinação em áreas menos urbanizadas aumentou de forma considerável a probabilidade de aceitação de todas as vacinas disponibilizadas em comparação com áreas sem um centro de vacinação ou em áreas mais urbanizadas que têm um centro de vacinação^27^.

Os resultados da análise de *clusters* sublinham desigualdades significativas na cobertura vacinal contra o HPV em Minas Gerais, destacando que os fatores socioeconômicos, o acesso à saúde, a urbanização e a aquisição de informações desempenham papéis determinantes na adesão à vacina HPV. Nesse sentido, o processo de urbanização, aliado ao melhor *status* socioeconômico do indivíduo, possibilita mais acesso aos serviços de saúde. Possibilita também maior acesso a informações sobre importância da prevenção das infecções associadas ao HPV e os benefícios da vacinação, o que, consequentemente, reflete-se em maior adesão à vacinação e aumento da CV.

Neste estudo, constatou-se menor percentual de municípios do estado de Minas Gerais com o alcance de meta de CV para a vacina HPV no sexo masculino. Identificou-se, ainda, que alta urbanização, menor vulnerabilidade social e maior acesso aos serviços de saúde são fatores que podem impactar positivamente a adesão à vacina contra HPV pelos adolescentes.

Apesar de ter havido avanços em alguns municípios com condições mais favoráveis, a baixa CV entre meninos, a discrepância entre as doses e as fragilidades estruturais de saúde pública em áreas vulneráveis apontam para a necessidade urgente de intervenções direcionadas para o público elegível para a vacinação contra o HPV, considerando os determinantes sociais.

A maior vulnerabilidade observada nos *clusters* 4 e 5 revelou a influência de barreiras sociais e logísticas, especialmente em regiões com maior extensão territorial e menor densidade populacional. Já os resultados positivos nos *clusters* 1 e 3 reforçam que investimentos em infraestrutura de saúde, políticas sociais e estratégias de mobilização comunitária podem aumentar significativamente a CV.

Com base nessas evidências, recomenda-se a priorização de municípios vulneráveis em campanhas de conscientização, melhorias na logística de vacinação e ampliação da ESF em regiões remotas. Além disso, é essencial o monitoramento contínuo da hesitação vacinal e o desenvolvimento de políticas públicas que integrem ações educativas, redução de desigualdades sociais e acesso universal à imunização.

Neste estudo, as principais limitações estão relacionadas à utilização de dados secundários para o cálculo de CV da vacina HPV, indicadores socioeconômicos, acesso à saúde e taxa de urbanização. Além disso, por se tratar de um estudo ecológico, os resultados encontrados não significam, obrigatoriamente, que a mesma associação aconteça em nível de indivíduos. Todavia, reforça-se o uso de metodologia rigorosa, e destaca-se a importância do uso dos modelos multivariados espaciais e de pesquisas que relacionem a CV com indicadores socioeconômicos e variáveis geográficas, possibilitando o melhor planejamento de ações direcionadas para a reconquista das CV.

Pode-se concluir que os resultados deste estudo reforçam a importância de abordagens intersetoriais para garantir a equidade em saúde, contribuindo para a ampliação da cobertura vacinal contra o HPV e a redução da incidência de doenças relacionadas no estado de Minas Gerais, Brasil.
